# “It goes against the grain”: A qualitative study of the experiences of parents’ administering distressing health‐care procedures for their child at home

**DOI:** 10.1111/hex.12532

**Published:** 2017-02-14

**Authors:** Gemma Spiers, Bryony Beresford

**Affiliations:** ^1^ Social Policy Research Unit University of York York UK

**Keywords:** children, children's health care, distress, health‐care procedures, parents, qualitative

## Abstract

**Background:**

Parents caring for children with complex and long‐term conditions at home take on responsibility for technical health‐care procedures that may cause their child distress. Little evidence exists about parents’ experience of this specific aspect of their caring role.

**Aims:**

To explore and understand parents’ experiences of administering distressing health‐care procedures as part of caring for their child at home.

**Design:**

An explorative qualitative study.

**Methods:**

A purposive sample of parents who were currently carrying out, or had previously carried out, health‐care procedures they thought their child found distressing was recruited. Data were collected using in‐depth interviews and analysed thematically.

**Findings:**

Administering these procedures was not just a clinical task. That the procedures caused distress for the child meant there were additional issues to consider and address. A major issue for parents was being able to prevent or minimize their child's distress, which in turn was closely linked to parents’ own emotional discomfort in the situation. Parents also had to manage their child's physical and verbal resistance, their own emotional discomfort during the procedure, and the presence and reaction of siblings in the home. The types of support that were valued by parents included advice about managing their child's distress and resistance, occasional assistance with procedures, addressing the emotional aspects of the role, and adequate training and on‐going supervision.

**Conclusion:**

The “added” challenges of assuming this responsibility have implications for the support of parents caring for ill children at home.

## 1 INTRODUCTION

Once discharged from hospital, children with complex or long‐term health conditions require on‐going support in the community. In caring for and managing their child's condition outside hospital, parents hold a multifaceted role and take on a range of responsibilities.^1^, ^2^, ^3^ This includes the administration of health‐care procedures, some of which may cause the child distress.^4^, ^5^ Few studies have examined parents’ experiences of assuming this responsibility. As a result, little is understood about any associated support needs.

## 2 BACKGROUND

Parents of children with complex or long‐term health conditions are often responsible for technical health care, including, for example, passing nasogastric tubes,^5^, ^6^ anal dilation following surgery,^7^, ^8^ tracheotomy care^9^ or managing infusion pumps for children with sickle cell disorder.^10^ Past studies report that because these procedures can be distressing for the child, parents may find this responsibility emotionally difficult^5^, ^6^, ^7^, ^11^ or decline it entirely.^4^, ^12^ However, existing evidence is very limited and restricted to studies with a broader focus on the parents’ experiences of caring.

Being responsible for administering distressing health‐care procedures is just one component of the caring role a parent may assume.^1^, ^3^ However, the potential implications are significant. For example, the emotional impact of this responsibility and observing their child's distress^5^, ^6^, ^7^, ^11^ may present support needs. We know that hospital staff find carrying out distressing procedures to be a source of stress, and peer support and supervision are important for easing this.^13^ It is, therefore, reasonable to argue that parents may have similar support needs.

Potential consequences for the parent‐child relationship have also been suggested,^7^, ^14^ although current evidence is inconclusive. For example, a small‐scale study of the effects of invasive anal treatment reported that around a third of parents observed a negative impact on their relationship with their child.^8^ Kirk et al.'s^5^ study of parents undertaking technical care tasks described parents as “being agents of pain rather than providers of comfort and protection” (p. 460), hinting at a conflicted parenting role. Others have noted a transitory suspension of the usual parental roles of protector and comforter. Both Callery^14^ and Tong et al.^6^ describe instances of parents “detaching” emotionally when passing their child's nasogastric tube or restraining their child during a painful procedure^.^ Beyond this, there is little detailed evidence about how parents make sense of and adapt to this responsibility, its perceived impact and what, if any, support they require.

English health‐care policy encourages children's health care to be delivered closer to home.^15^, ^16^, ^17^ This reflects a UK‐wide approach to shift care from hospitals to the community.^18^ This, combined with the growing number of children with life‐limiting conditions,^19^ means it is likely that more parents will assume responsibility for their child's health‐care at home. Inevitably, this will include the administration of health‐care procedures that potentially cause the child distress. It is, therefore, important to address this gap in evidence and better understand parents’ experiences of this specific aspect of care, the implications of the situation and the associated support needs. Such evidence is especially pertinent to children's nurses, who play a core role in supporting parents caring for ill children at home.^2^

To address this gap in evidence, this study aimed to understand parents’ experiences of carrying out nursing and health‐care procedures that caused their child distress, to explore the perceived impact on them, their child and their family, and to identify support needs. This study reports findings from the study.

## 3 METHODS

An explorative, qualitative study design was used, which is appropriate when investigating topics with limited existing evidence. In‐depth interviews were used as these are effective at eliciting rich information for under‐researched topics.^20^

### 3.1 Participants and recruitment

We aimed to recruit no more than 20 parents to allow in‐depth exploration of the topic. There were two criteria for study inclusion. First, the parents must have had current or past experience of carrying out technical health‐care procedures that *they* thought their child found distressing. Second, the child was aged approximately 10 years or less. In terms of exclusion, we purposefully avoided recruiting older children; issues relating to puberty may have added another layer of complexity to the topic that could not be accommodated within this project.

No further criteria were used, but we tried to ensure a range of factors were represented in the sample (see Box 1). Participants were recruited via adverts placed on websites and newsletters of charitable organizations, and social media (see Box 2). The adverts contained a web link to further information and an online form which could be used to express interest in participating. Parents could also contact the researchers directly. Those who expressed interest were contacted by the researchers to discuss the possibility of taking part in the research.

Box 1 **Purposive sampling**

1

**Table:** 

Inclusion criteria: Parents who are currently, or have in the past, carried out procedures that they think their child finds distressing Parents whose child is aged approximately <10 years.
Additional factors represented in the sample: A range of ages between birth and approximately 10 years Children with and without cognitive impairments (indicated by parental reported level of difficulty with learning and communication) Children with both sudden onset conditions and conditions present from birth Parents who did and did not share responsibility for the procedures with a family member Both long and short durations of holding responsibility for the procedures (defined here as less or more than 6 months)

Box 2 **Websites and organizations where the recruitment advertisement was placed**

1

**Table:** 

WellChild closed Facebook page, local branches and email list
Together for Short Lives newsletter
Children's Heart Association website
Cerebra Facebook page
CLIC Sargent Facebook page
Lagan's Foundation Facebook page
Tweeted from SPRU and WellChild Twitter accounts
Distributed via the Social Policy Research Unit's Parent Consultation Group

### 3.2 The sample

Nineteen primary caregivers were recruited. These were mainly mothers. A range of diagnoses, family composition, types of procedures, duration of responsibility for the procedures and cognitive ability of the child was represented (see Table 1). Recruitment was UK‐wide with a broad geographical spread within the sample. A brief description of the procedures that parents undertook for their child is provided in Table 2. We also recruited a subsample of secondary carers (usually fathers). However, this paper focuses on the findings from the primary caregivers.

**Table 1:** Sample description of primary caregivers interviewed

Total	19
Mothers	18
Fathers	1
Single	2
Married or living with a partner	17
Age range	27‐53 years^a^
Age range of children at the time of being recipient to the nursing/health‐care procedures	3 months to 11 years^b^
Procedures parents carried out	
Inserting nasogastric tube	9
Changing and routine care of tracheostomy tubes	5
Insertion or changing of gastrostomy feeding tube or button	5
Finger/thumb pricks	4
Other nasal procedures such as inserting nasal prongs and cannulas, nasal suctioning	4
Injections	2
Changing of dressings	2
Oral suctioning	2
Jejunostomy	1
Bladder manipulation	1
Colostomy care	1
Suppositories	1

*Conditions of children represented in the sample (some generalized if very rare):* brain damage; cancer/leukaemia; cerebral palsy; congenital condition affecting the urethra and rectum; congenital heart conditions; low immunity; lung disease; neurodegenerative conditions; rare chromosome conditions; undiagnosed.

a Data not available for two participants; one participant described her age as “early sixties.”

b One child had just turned 11 years of age at the time of recruitment and was thus included in the study.

**Table 2:** Description of the procedures parents undertook for their child

*Inserting nasogastric tube* Passing a thin tube through the nasal cavity into the stomach
*Changing and routine care of tracheostomy tubes* A tracheostomy is a surgically placed tube that enters the airway through a hole in the throat. The procedures associated with this that parents carried out were reinserting the tube when required, suctioning (ie removing secretions from the airway), keeping the tube clean and removing and applying dressings that keep it in place.
*Care and changing of gastrostomy feeding tube or button* A gastrostomy is a surgically placed tube/button that enters the stomach through a hole in the abdomen. The procedures associated with this that parents carried out were reinserting it when it came out, turning the tube whilst inserted (to prevent it fusing with skin tissue), keeping it clean and removing and applying the dressings that keep it in place.
*Finger/thumb pricks* Pricking of finger or thumb to elicit blood, which is then tested (eg to monitor blood sugar)
*Inserting nasal prongs/cannulas* Insertion of tubes into the nasal cavity
*Injections* Needle injection of medication into, for example, the child's thigh
*Changing of dressings* Removal and application of dressings (eg facial dressings for nasogastric tubes)
*Oral/nasal suctioning* A suction tube placed in the child's mouth/nose to remove secretions
*Jejunostomy care* A jejunostomy is a surgically placed tube that enters the jejunum through a hole in the abdomen. The procedures associated with this that parents carried out were reinserting it when it came out, keeping it clean and removing and applying the dressings that keep it in place.
*Bladder manipulation* Manual manipulation of bladder
*Colostomy care* Cleaning of colostomy site and changing of colostomy bag
*Suppositories* Medication inserted into the rectum

### 3.3 Data collection

Interview schedules ensured consistency and comprehensiveness of topic coverage across all interviews (see Box 3, column A). The topics explored in the interview were informed by the research questions and objectives, consultation work with children's community nurses conducted to support the bid for funding this research, and findings from existing research. Draft topic guides were shared and discussed with our research unit's permanent consultation group of parents of children with complex needs. Interviews took place either face to face or via telephone depending on parents’ preference. They typically lasted around one hour (range 39‐145 minutes). Interviews were audio‐recorded with permission and transcribed verbatim. The data were collected during 2014‐2015.

Box 3 **Topics, overarching themes and refined themes**

1

**Table:** 

Column A Topics covered in interviews, data from which fed into overarching themes/mind maps	Column B Initial and overarching themes developed from the pen portraits around which the data were organized and displayed in the mind maps. One mind map was used for each theme	Column C The themes after refinement through the thematic analysis, and the mind‐maps/overarching theme (in brackets) from which these were drawn
Background and circumstances of the parent, child and family Preparing for and undertaking the procedures Experience of undertaking the procedure Managing their child's reactions Perceived impact Support needs Advice and suggestions for others	(A) How did parents come to have responsibility for the procedure (B) Training and preparation for the procedures (C) What is it like for the parent to carry out these procedures (D) Perceived acceptability of having this responsibility (E) Managing the situation (self, child, siblings, environment, spousal divisions of responsibility) (F) Implications for the parent‐child relationship (G) On‐going support	Emotional experiences and responses (C, D, E) Making sense of the role (A, C, D) Changes in experience over time (C, D, E) The added demands of the role (C, E) Associated support needs (B, G, C, E) *(Note: data from theme F are not presented in this paper)*

### 3.4 Ethical considerations

This was a sensitive topic on which to undertake research, and strategies were put in place to pre‐empt and address this. First, all information conveyed to participants made it clear that the interview could be pause or stopped at any time. Second, the structure of the topic guide ensured participants were eased into difficult topics and that interviews ended on less sensitive issues. Third, participants were given a leaflet containing details of local and national support and advice organizations for parents of children with complex health‐care needs. Fourth, the researchers anticipated that due to the topic, some participants may become upset during interviews, and prepared a strategy to manage this prior to embarking on fieldwork. This involved sensitively acknowledging the participants’ distress, asking the participant whether they would like to pause or end the interview, and only continuing if and when the participant felt ready. Only a minority of participants became upset, but all wanted to continue with the interview. Informed consent was obtained from all parents, and all were given £20 as a thank you for their time. Ethical approval was obtained from the University's Social Policy & Social Work departmental ethics committee.

### 3.5 Data analysis

A thematic approach^21^ to data analysis was taken. It comprised a four‐stage process. First, transcripts were read and re‐read, with extensive notes written to facilitate familiarization with the data and to begin identifying emerging themes in parents’ accounts. We then prepared “pen portraits” of each interview which yield a condensed account whilst retaining the complexity, context and narrative. These pen portraits included participant quotes (referenced with transcript page numbers for transparency and tracking), and a series of bullet points to summarize the key themes of the interview. Portraits were typically 3 to 5 pages long, and text was organized under five categories: *the participants’ circumstances, how they came to have responsibility for carrying out the procedures, parents’ experiences of the procedures, perceived impact* and *support needs*. The themes and narratives of the pen portraits were then collated into a set of overarching themes (see Box 2, column B) and displayed visually, with summaries of supporting data, using mind maps. Mind maps are a tool for visually displaying themes and subthemes and the connections between them. Mind Genius^®^ software was used to support this process. Using the mind maps and pen portraits, we then undertook a thematic analysis.^21^ This involved writing analytical notes comprising thick layers of description about the themes, patterns and typologies. As part of this, we compared groups and examined cases to seek explanations for the findings. These notes were then reworked and refined until the team was satisfied the analytical process was complete and the account produced comprehensive and accurate. This resulted in the refined themes reported in this paper (see Box 3, column C).

### 3.6 Validity and rigour

Validity and rigour in this study were supported through numerous strategies. At the stage of recruitment, clear information was given to ensure we sampled relevant “key informants” in our purposive sample.^22^ At data collection, an interview schedule ensured the topics explored were consistent across interviews. Throughout analysis, [GS] and [BB] regularly discussed the data and our interpretations, cross‐checking emerging themes to enhance reliability of interpretation.^23^ Data analysis tools (pen portraits, mind maps) included systems by which summarized/reduced data were referenced back to the raw data. Towards the end of analysis, a small group of stakeholders (nurses, paediatric psychologists, play therapists, voluntary sector representatives and parents) met to discuss and reflect on the emerging findings. This discussion, facilitated by the research team, fed into final data interpretation, thus enhancing its validity. Finally, methods are reported clearly here for purposes of transparency and understanding.^24^

## 4 FINDINGS

Findings are presented around five key themes (see Box 3, column C):

- emotional experiences and responses
- making sense of the role
- changes in experiences over time
- the added demands of the role
- associated support needs.

We use quotes throughout for illustrative purposes.

### 4.1 Emotional experiences and responses

Parents described negative emotional responses to being responsible for carrying out procedures that caused some observable or inferred distress for their child. They described it as something that was “upsetting,” “awful,” “really tough,” “unpleasant,” “horrific,” “horrid,” “horrible,” “horrendous,” “stressful,” “not nice,” “traumatic” or something they “hated”. There were feelings of guilt, linked to the conflict between being a parent, and therefore a protector and comforter, and having to do “horrible” things to one's child:

Oh it, it's the guilt all the time, because it goes against the grain; you're there to look after your child and you're doing all these things so that you can look after your child, but at the same time it, they're not nice things, they're, they're quite horrible.

(Interview 3: Mother, nasogastric tube, finger pricks)

Not all felt this way: for two parents, being responsible for procedures that caused their child distress either presented little emotional burden or did not occupy a significant emotional “space.” One mother, who gave thumb pricks to her child, indicated that whilst she did not like doing this, it was not something that she typically struggled with. Other aspects of her child's care were more “significant,” and, because her son had come close to dying on more than one occasion, she was willing to take on this component of care: “you don't care what it means to do anything extra cos you're just glad to have them” (Interview 6, Mother, thumb pricks). Another mother's account indicated that it was the *responsibility* for life‐preserving care (tracheostomy tubes) that was the most demanding part of her caring role.

Where there *was* emotional discomfort, or unease, parents reflected on this as something that was amplified in the moment of carrying out the procedures and they described the coping strategies they used. First, they described efforts to not to dwell too closely on their own emotions or their child's reactions. This was reflected in language such as “not thinking about it too much” or “blocking.” Such “blocking” of emotions and thoughts allowed parents to focus on carrying out the procedure:

I think if you sit there and think about it you won't, you won't do it

(Interview 5: Mother, suctioning, gastrostomy tube)

Second, “temporary” roles were adopted. Parents reported going into “nurse mode,” being a “different” person or having to “get out of the mother zone” so as not to engage with their maternal feelings about the procedures.

These were the ways parents handled their discomfort *in* the moment of carrying out the procedures. Separate to this, when reflecting on their experiences during the interview, some parents reconciled their responsibility for carrying out distressing procedures by making sense of their role in the context of perceived benefits and choice. We go on to describe this next.

### 4.2 Making sense of their role

There were two ways by which parents made sense of having to do something that caused their child distress.

First, for some parents, being responsible for these procedures was seen as a trade‐off: it allowed them to achieve other outcomes that would not be possible if health staff carried out the procedures. For example, some highlighted avoiding trips to and time spent in hospital. Others felt that their child was less distressed if they, as opposed to a health professional, performed the procedures:

I just thought well if it was me I'd much rather my mum did something like that than someone I've never seen before leaning over me and poking a tube up my nose, I'd rather it was someone I trusted

(Interview 16: Mother, nasogastric tube, suctioning)

This notion of “trustworthiness” referred to in the quote above may have been linked to a perception that, as their child's protector, they could also be a source of comfort during procedures:

So even if she didn't fully understand, she could hear my voice and it was mummy and it was all about the reassurance.

(Interview 4: Mother, various procedures)

Thus, even though they disliked having to do the procedures, the perceived benefits to the child and/or family were greater or prioritized. Indeed, some proactively made choices to administer the procedures, in place of health staff, to achieve these trade‐offs.

Second, some parents believed they had no choice about being responsible for these procedures. This perceived absence of choice appeared to be used by some parents as a way of reconciling their feelings about the role:

I really don't like doing it and I don't like knowing that that's got to be done that day but it's, it's just a part of what needs to be done so, you know, there's no choice really.

(Interview 6: Mother, dressings, thumb pricks)

### 4.3 Changes in experience over time

For most, the experience of being responsible for carrying out procedures that caused their child distress changed over time: objection transformed to acceptance, anxiety and doubt were replaced with a sense of competence, and what was once an extraordinary part of their role became “normal.” This acceptance was reflected in the way parents described how they still disliked carrying out the procedure, followed by the caveat of “but.” For example:

I don't like doing it but, as I say, better me doing it than a stranger

(Interview 2: Mother, injections)

I didn't like it but needs be must

(Interview 5: Mother, tracheostomy, gastrostomy, suctioning)

It is upsetting when you're having to, to do that, you know, and we're upset, and it's because of something you're doing to her. But yeah, you've just got to

(Interview 12: Mother, finger pricks)

This acceptance of the role was particularly observed in those carrying out procedures that were not time‐limited. Thus, the long‐term inevitability of the responsibility may have shaped such acceptance. There was also evidence that carrying out the procedures regularly enhanced familiarity with, and thus acceptance of, them. For some, carrying out the procedures ceased being an anomalous part of their role, with descriptions of these becoming a “normal” component of their caring responsibility as a parent over time. For example, one parent described the changing and care of her son's tracheostomy tube as something that became “as normal to me as changing his nappy” (Interview 1).

A greater sense of competence in carrying out the procedures, developed over time, was also described by many. This was linked to the child becoming less distressed (in some cases as a result of the way parents managed this or because the child better understood what was happening with age), or more compliant. Whilst the child's increased understanding of what was happening was implicated in the lessening of their distress (and thus parents’ sense of competence), there was no evidence that the child's age played a role in how quickly parents adapted to this responsibility.

For some, there was a desire to become more confident and competent in carrying out the procedures because it was felt this would make it less distressing for the child:

I think the first nurse that I ever saw do it was incredibly quick and it made such a difference seeing it done at that speed that you could see it was just a moment's discomfort [for child]. So I think it was just a case of me saying that I need to get as good as that and then it'll be OK

(Interview 16: Mother, nasogastric tube, suctioning)

This growing sense of acceptance, competence and/or perceived normality of performing the procedures was linked by parents to a lessening of emotional burden. However, not all the parents reported this and, for a minority, it was clear there was still, or had been, a degree of emotional struggle. This was observed among parents carrying out procedures for a time‐limited period, and those where it was an on‐going responsibility. Among parents where the procedures were time‐limited, it is possible; this meant that parents did not have the time or opportunity to adjust to the responsibility and/ or did not engage in the emotional work of the process of adjustment and adaptation to the role. Where responsibility for procedures was on‐going, emotional struggles were linked to a perceived absence of, and need for, professional support:

I think every now and again, even if they just offered to come out and assist with it or, they're just like once they know you can do it you're just left to get on with it.

(Interview 14: Mother, gastrostomy tube)

Regardless of whether the emotional burden lessened over time, there were still other demands that parents had to contend with *because* the procedures caused their child distress. In the next section, we describe what might be considered as these “added challenges.”

### 4.4 The added demands

The procedures that parents carried out were not simply a clinical task. Because the procedures elicited some form of distress in the child, parents had to manage this. To manage, or prevent, distress, various strategies were tried, mostly initiated by parents themselves but in a few cases with the guidance of health staff.

Some strategies sought to distract the child from the anticipated or actual pain or discomfort. Other strategies parents described included getting the child involved to allow them some control over what was happening, bargaining, having a known and familiar routine, and explaining and reassuring. The perceived success of these strategies varied, and whilst some were confident in managing their child's distress and felt it was best guided by parents, others found it a challenge:

We tried to encourage him and then we kind of, like everything you're not meant to do, we tried to bribe him… But … he knows his own mind and even from a young age he knew, he was not having it, it didn't work.

(Interview 9: Mother, nasal cannulas)

There could also be physical resistance from the child, requiring some form of holding or restraint. Many developed their own ways of doing this, but restraint itself was another source of unease for parents. One mother noted how she had become “an expert at dodging arms” as restraint had never been suggested by health staff as an option, and nor was it something she was comfortable with. Restraining a child *and* undertaking the procedure could be a two‐person job and thus was a further challenge for those acting alone.

Some of those interviewed had other children and managing their presence during the procedure, and their reactions to their upset brother or sister, presented parents with another situation to manage. Some insisted on carrying out the procedures with only them and their child present, with other children occupied by another family member so that they did not get “in the way” or so that they could not see what was happening:

it's not the sort of thing that I would, that she would need to see, me doing something to upset her brother

(Interview 8: Father, nasogastric tube)

For single parents, or those who did not have another family member in the house at the time, the presence of siblings was necessary or inevitable. There were reports of making the best of this situation, by getting the sibling involved to “demystify” the procedure, but also a sense of guilt from parents that their other children may end up helping them. A minority recalled initial concerns about how siblings would or did respond to seeing their brother or sister upset. However, none felt this had become problematic in the long‐term, and parents believed they had successfully explained what was happening to their other children.

So far, the findings reported have revealed how carrying out distressing health‐care procedures means parents are managing a complex situation that extends beyond the technical delivery of the procedure alone. Next, we report the ways in which parents wanted to be supported with this responsibility.

### 4.5 Parents’ support needs

Parents’ experiences of being supported with this responsibility varied. Some described on‐going contact with ward and/or community nurses, and others reporting little to no input after the initial training period. The input from health‐care staff, in particular nurses, which parents valued or did *not* have but desired, reveals four areas of support.

The first related and responded to the fact that the procedures caused their child distress. As such, there was an expressed need for advice about ways of managing their child's distress, and ways of managing, and assistance with, restraining the child. One parent also highlighted useful advice she had received about distracting her other children.

Second, for some parents, there was a desire for an occasional break from the “nursing” role, with the responsibility being temporarily assumed by health‐care practitioners:

it would have just been, been nice that other people could put [the tube] back in… I would rather be a mum than be a medically qualified doctor/nurse.

(Interview 20: Mother, jejunostomy tube)

Third, the importance of practitioners recognizing and addressing the emotional demands of the role, and any associated support needs, was stressed even among parents who believed they had adjusted to the responsibility. Opportunities for peer support, being asked by their child's nurse how they, as a parent, feel about doing the procedures, and recognition from health‐care teams of the responsibility parents have when taking on these procedures were all ways of providing emotional support. Some felt such support would be more beneficial, or critical, in the earlier stages of being responsible for the procedures, whilst others did not identify a point at which it would be most useful, implying it would be valued at any time.

The fourth area of support concerns parents’ anxieties about performing the technical aspects of the procedure correctly. This type of support thus extended beyond the fact that these procedures were distressing, and the associated demands this created. This could be achieved through paced training at the stage of learning the procedure that allowed the parent to grow in confidence and address anxieties, opportunities to refresh training later through intermittent supervision and observation from nurses, the provision of information about carrying out the procedures and, importantly, access to a ward or community nurse once at home to call for advice if needed.

## 5 DISCUSSION

First and foremost, these findings show that health‐care procedures, such as inserting nasogastric tubes, care and changing of gastrostomy and tracheostomy tubes, giving injections and finger pricks, and inserting nasal cannulas and prongs, are not just clinical tasks for parents. The distress they can cause the child means that the task expands to encompass their own experiences of emotional discomfort and adjusting to that, the management of the child's distress and resistance, and consideration about the role and presence of siblings. Importantly, these demands and the circumstances in which they take place are likely to be hidden from nursing staff and other health‐care professionals, especially those that are ward based. This “invisibility” has implications for how parents’ support needs are recognized and met.

Nurses, especially those with a community function, are expected to play an important role in supporting parents of children with on‐going health needs at home,^16^ yet evidence suggests a mixed picture about the extent to which this is achieved. Whilst evaluations of specific children's community nursing services have been positive (eg^4^, ^25^), other studies indicate that parents may not always be adequately supported or given sufficient breaks in their caring role.^26^, ^27^ We found a similar pattern of evidence here: some parents felt sufficiently supported as they assumed responsibility for health‐care procedures, whilst others had little or no on‐going support or input. The mixed reports of nursing support and assistance to parents, both here and in other studies, may reflect an uneven provision of community nursing that has been evidenced in earlier studies.^2^, ^28^

In the context of the responsibility parents may assume for carrying out distressing procedures at home, nursing input may play a particularly critical role, especially in terms of providing emotional support and occasional assistance. In terms of the latter, this was linked to a desire to (greater) experience being a parent, as opposed to a proxy health professional. However, whilst nurse input may at times need to be “hands on,” we also found evidence of a relatively simple way of supporting parents. This was in the form of professionals offering recognition of the responsibilities and impact of taking on the administration of these procedures. Other studies report that parents can perceive professionals as not valuing, or recognizing, the role they play in the care of their child.^1^ The findings from this study reiterate that this, in itself, is a valued way of supporting parents.

Parents’ views on how they can be best supported to assume responsibility for administering distressing procedures reflect, to some extent, findings reported elsewhere about the role of nurses supporting families of ill children in the community. For example, the importance of emotional and information support, and being able to contact a nurse to seek reassurance, has been evidenced previously.^4^, ^25^, ^29^, ^30^ However, our findings about being supported with aspects relating to the child's distress are novel. They raise new questions about who is best placed to provide this support and how. For example, there may be a role for play therapists and paediatric psychologists to work alongside nurses when parents are being initially trained in procedures or, subsequently, raise concerns with respect to this. Previous work, however, shows these specialists are not routinely part of health‐care teams for children with complex health conditions.^2^

The findings also have implications for wider debates around management of child distress and resistance in health care. Guidance has been developed in the UK,^31^ but this is for health professionals rather than parents. It is clear, however, that parents may also administer procedures that cause the child distress, and highlights an important gap in the governance of children's health and nursing care in the community. The emotional experience of parents was closely tied to the child's response to the procedure. Thus, the importance of being able to manage the child's distress is not just about positive practice for the child, but also has implications for parents’ emotional wellbeing. Studies have shown the importance of supervision and support for health staff carrying out procedures that cause children distress.^13^ Such supervision and support are, arguably, equally important for parents.

Effective management of child distress is also critical as it minimizes the need for restraint, something which parents reported sometimes having to do and which could cause both them and their child further distress. In the context of procedures carried out by staff, where the “holding” of children may be carried out by staff or parents, Bray et al.^32^ question the ethics of restraint. The same question might be asked of situations where parents are restraining children *and* carrying out procedures. Again, this underlines the value of supporting parents to prevent, minimize or adequately manage their child's distress.

A final reflection on these findings concerns the theme of choice. Some parents perceived they had choice in carrying out these procedures, whilst others did not. A reliance on parents to provide care has been noted previously,^1^ and it raises questions about the extent to which the UK's care closer to home policy is adequately supported in practice.

### 5.1 Study strengths and limitations

Given the lack of existing evidence, the in‐depth, exploratory approach is a major strength. It is, however, important to note that we struggled to recruit “secondary carers,” usually fathers. This is not an unfamiliar experience^33^ but does mean the study has not been able to explore the experiences of others who may, in some way, be involved in administering health‐care procedures which distress their child. Whilst mother are typically the main carers for ill children, fathers’ views are important, especially in this context where parents may be negotiating responsibility for carrying out procedures, or where responsibility is shared. Finally, the self‐selecting sample presents a source of bias. However, a range of procedures and children's conditions were represented, thus minimizing the potential for these exploratory findings to be applicable only to particular groups of children or procedures.

## 6 CONCLUSION

Carrying out health‐care procedures that cause their child distress generates a unique set of support needs for parents, which, potentially, may be hidden from health‐care teams. There are implications for children's health‐care professionals about how to recognize and meet these needs.

## CONFLICT OF INTEREST

No conflict of interest has been declared by the authors.

## References

[1] Ward C , Glass N , Ford R . Care in the home for seriously ill children with complex needs: a narrative literature review. J Child Health Care. 2015;19:524–531. 24982427 10.1177/1367493514538327

[2] Parker G , Spiers G , Gridley K , et al. Evaluating models of care closer to home for children and young people who are ill: main report. NIHR Service Delivery and Organisation Programme, Southampton;2011.

[3] McCann D , Bull R , Winzenberg T . The daily patterns of time use for parents of children with complex needs: a systematic review. J Child Health Care. 2012;16:26–52. 22308543 10.1177/1367493511420186

[4] Spiers G , Parker G , Gridley K , Atkin K . The psychosocial experience of parents receiving care closer to home for their ill child. Health Soc Care Community. 2011;19:653–660. 21623985 10.1111/j.1365-2524.2011.01008.x

[5] Kirk S , Glendinning C , Callery P . Parent or nurse? The experience of being the parent of a technology‐dependent child. J Adv Nurs. 2005;51:456–464. 16098162 10.1111/j.1365-2648.2005.03522.x

[6] Tong A , Lowe A , Sainsbury P , Craig JC . Parental perspectives on caring for a child with chronic kidney disease: an in‐depth interview study. Child Care Health Dev. 2010;36:549–557. 20412147 10.1111/j.1365-2214.2010.01067.x

[7] Cavet J . Children and young people with a hidden disability: an examination of the social work role. Br J Soc Work. 2000;30:619–634.

[8] Diseth TH , Egeland T , Emblem R . Effects of anal invasive treatment and incontinence on mental health and psychosocial functioning of adolescents with Hirschsprung's disease and low anorectal anomalies. J Pediatr Surg. 1998;33:468–475. 9537559 10.1016/s0022-3468(98)90090-2

[9] DeRowe A , Fishman G , Leor A , Kornecki A . Improving children's cooperation with tracheotomy care by performing and caring for a tracheotomy in the child's doll—a case analysis. Int J Pediatr Otorhinolaryngol. 2003;67:807–809. 12791458 10.1016/s0165-5876(03)00065-x

[10] Atkin K , Ahmad WIU . Family care‐giving and chronic illness: how parents cope with a child with a sickle cell disorder or thalassaemia. Health Soc Care Community. 2000;8:57–69. 11560675 10.1046/j.1365-2524.2000.00211.x

[11] Sanders C , Bray L , Driver C , Harris V . Parents of children with neurogenic bowel dysfunction: their experiences of using transanal irrigation with their child. Child Care Health Dev. 2013;40:863–869. 24261461 10.1111/cch.12117

[12] Coyne IT . Partnership in care: parents’ views of participation in their hospitalized child's care. J Clin Nurs. 1995;4:71–79. 7704384 10.1111/j.1365-2702.1995.tb00014.x

[13] Lloyd M , Urquhart Law G , Heard A , Kroese B . When a child says ‘no’: experiences of nurses working with children having invasive procedures. Paediatr Nurs. 2008;20:29–34. 10.7748/paed2008.05.20.4.29.c8252 18547007

[14] Callery P . Paying to participate: financial, social and personal costs to parents of involvement in their children's care in hospital. J Adv Nurs. 1997;25:746–752. 9104670 10.1046/j.1365-2648.1997.t01-1-1997025746.x

[15] DH, DfES . National Service Framework for Children, Young People and Maternity Services. London: Department of Health, Department for Education and Skills; 2004.

[16] DH . NHS at Home: Children's Community Nursing Services. London: Department of Health; 2011.

[17] Monitor . Moving Healthcare Closer to Home: Summary. London: Monitor; 2015.

[18] Department of Health . Our Health, Our Care, Our Say: A New Direction for Community Services. London: Department of Health; 2006.

[19] Fraser L , Miller M , Aldridge J , McKinney P , Parslow R . Prevalence of life‐limiting and life‐threatening conditions in young adults in England 2000‐2010. Together for Short Lives; 2013.

[20] Ritchie J , Lewis J . Qualitative Research Practice. London: Sage; 2003.

[21] Braun V , Clarke V . Using thematic analysis in psychology. Qual Res Psychol. 2006;3:77–101.

[22] Emmel N . Sampling and Choosing Cases in Qualitative Research: A Realist Approach. London: Sage; 2013.

[23] Green J , Thorogood N . Qualitative Methods for Health Research. London: Sage; 2009.

[24] Mays N , Pope C . Assessing quality in qualitative research. Br Med J. 2000;320:50–52. 10617534 10.1136/bmj.320.7226.50 PMC1117321

[25] Callery P , Kyle RG , Banks M , Ewing C , Kirk S . Enhancing parents’ confidence to care in acute childhood illness: triangulation of findings from a mixed methods study of Community Children's Nursing. J Adv Nurs. 2013;69:2538–2548. doi:10.1111/jan.12141. 23560950 10.1111/jan.12141

[26] Whiting M . Impact, meaning and need for help and support: the experience of parents caring for children with disabilities, life‐limiting/life‐threatening illness or technology dependence. J Child Health Care. 2012;17:92–108. 23220736 10.1177/1367493512447089

[27] Kirk S , Glendinning C . Developing services to support parents caring for a technology‐dependent child at home. Child Care Health Dev. 2004;30:209–218. 15104576 10.1111/j.1365-2214.2004.00393.x

[28] Tatman MA , Woodroffe C . Paediatric home care in the UK. Arch Dis Child. 1993;69:677–680. 8285782 10.1136/adc.69.6.677 PMC1029653

[29] Smith L , Daughtrey H . Weaving the seamless web of care: an analysis of parents’ perceptions of their needs following discharge of their child from hospital. J Adv Nurs. 2000;31:812–820. 10759977 10.1046/j.1365-2648.2000.01339.x

[30] Kirk S , Glendinning C . Supporting ‘expert’ parents—professional support and families caring for a child with complex health care needs in the community. Int J Nurs Stud. 2002;39:625–635. 12100874 10.1016/s0020-7489(01)00069-4

[31] Duff AJA , Gaskell SL , Jacobs K , Houghton JM . Management of distressing procedures in children and young people: time to adhere to the guidelines. Arch Dis Child. 2012;97:1–4. 22082500 10.1136/archdischild-2011-300762

[32] Bray L , Snodin J , Carter B . Holding and restraining children for clinical procedures within an acute care setting: an ethical consideration of the evidence. Nurs Inq. 2015;22:157–167. 25053126 10.1111/nin.12074

[33] Macfadyen A , Swallow V , Santacroce S , Lambert H . Involving fathers in research. J Spec Pediatr Nurs. 2011;16:216–219. 21702882 10.1111/j.1744-6155.2011.00287.x

